# Effectiveness of inspiratory muscle training in patients with a chronic respiratory disease: an overview of systematic reviews

**DOI:** 10.3389/fspor.2025.1549652

**Published:** 2025-05-21

**Authors:** Rodrigo Torres-Castro, Saul Caicedo-Trujillo, Elena Gimeno-Santos, Ruvistay Gutiérrez-Arias, Xavier Alsina-Restoy, Luis Vasconcello-Castillo, Pamela Seron, Martijn A. Spruit, Isabel Blanco, Jordi Vilaró

**Affiliations:** ^1^ Department of Pulmonary Medicine, Hospital Clínic, University of Barcelona, Barcelona, Spain; ^2^ Fundació de Recerca Clínic Barcelona-Institut d'Investigacions Biomèdiques August Pi i Sunyer (IDIBAPS), Barcelona, Spain; ^3^ Department of Physical Therapy, Faculty of Medicine, University of Chile, Santiago, Chile; ^4^ Centro Médico Familiar Integral y Especialidades Diálisis “LA MARISCAL”, Instituto Ecuatoriano de Seguridad Social, Quito, Ecuador; ^5^ Programa de Doctorat-Facultat Ciències de la Salut Blanquerna, Universitat Ramon Llull, Barcelona, Spain; ^6^ Barcelona Institute for Global Health (ISGlobal), Barcelona, Spain; ^7^ Biomedical Research Networking Center on Respiratory Diseases, CIBERES, Madrid, Spain; ^8^ Departamento de Apoyo en Rehabilitación Cardiopulmonar Integral, Instituto Nacional del Tórax, Santiago, Chile; ^9^ Exercise and Rehabilitation Sciences Institute, Faculty of Rehabilitation Sciences, Universidad Andres Bello, Santiago, Chile; ^10^ INTRehab Research Group, Instituto Nacional del Tórax, Santiago, Chile; ^11^ Departamento de Ciencias de la Rehabilitación, Facultad de Medicina, Universidad de La Frontera, Temuco, Chile; ^12^ Centro de Excelencia CIGES, Universidad de La Frontera, Temuco, Chile; ^13^ Department of Research and Development, Ciro, Horn, Netherlands; ^14^ Department of Respiratory Medicine, School of Nutrition and Translational Research in Metabolism, Faculty of Health Medicine and Life Sciences, Maastricht University Medical Centre, Maastricht, Netherlands; ^15^ ERN-LUNG (European Reference Network on Rare Respiratory Diseases), Spain; ^16^ Blanquerna School of Health Sciences, Global Research on Wellbeing (GRoW), Universitat Ramon Llull, Barcelona, Spain

**Keywords:** inspiratory muscle training, chronic respiratory diseases, overview, exercise tolerance, symptoms

## Abstract

**Introduction:**

There has been inconclusive findings regarding the effectiveness of inspiratory muscle training (IMT) in chronic respiratory diseases (CRDs). Our objective was to determine the effectiveness of IMT on exercise tolerance, maximum respiratory pressure, lung function, symptoms and quality of life in different CRDs.

**Methods:**

We conducted an overview of systematic reviews (SRs) in adults with CRDs who underwent IMT. We reviewed five databases in March 2025. We chose the most comprehensive SRs to report on the analysed outcomes.

**Results:**

Twenty-three SRs were included. In chronic obstructive pulmonary disease (COPD), IMT increased the six-minute walk distance (6MWD) by 35.7 m (95% CI 25.7, 45.7), maximum inspiratory pressure (MIP) by 10.9 cmH_2_O (95% CI 8.0, 13.9). In asthma, IMT increased the forced expiratory volume in the first second (FEV_1_) by 3.3%pred (95% CI 1.4, 5.1), forced vital capacity (FVC) by 4.1%pred (95% CI 1.0, 7.3), MIP by 21.9 cmH_2_O (95% CI 15.0, 28.8), and dyspnoea was reduced (standard mean difference −0.8, 95% CI −1.3,−0.2). In obstructive sleep apnoea (OSA), IMT increased MIP by 29.6 cmH_2_O (95% CI 6.0, 53.1). In pulmonary hypertension (PH), IMT increased 6MWD by 39.0 m (95% CI 20.7, 57.4), MIP in 21.2 cmH_2_O (95% CI 11.3, 31.1), maximum expiratory pressure by 14.4 cmH_2_O (95% CI 6.9, 21.9), and dyspnoea was reduced by 0.5 (95% CI 0.1, 0.9) in modified Medical Research Council scale. In lung resection (LR), IMT increased MIP by 8.1 cmH_2_O (95% CI 1.3, 14.9). In bronchiectasis, IMT increased MIP by 6.1 cmH_2_O (95% CI 1.4, 10.8). Overall, the most consistent effect of IMT across different CRDs was an increase in MIP.

**Conclusion:**

IMT improved several clinically relevant outcomes, including MIP, exercise capacity, and dyspnoea in different CRDs. However, the limited evidence for certain outcomes and populations highlights the need for further high-quality studies.

## Introduction

Chronic respiratory diseases (CRDs) are a major public health problem, with nearly 4 million deaths in 2017, accounting for 7% of all deaths worldwide (1). Some of the most common CRDs are asthma, chronic obstructive pulmonary disease (COPD), and occupational lung diseases (2).

Individuals with CRDs often experience limitations in exercise tolerance due to a combination of factors, including alterations in ventilation and gas exchange, cardiovascular comorbidities, and peripheral muscle abnormalities (3). They also commonly have inspiratory muscle dysfunction, which is linked to symptoms such as dyspnoea and reduced exercise capacity (4). The causes of respiratory muscle dysfunction in CRDs patients are diverse and vary depending on the specific disease. For example, static lung hyperinflation in patients with COPD or asthma (5), and reduced lung compliance in patients with interstitial lung diseases (ILD) (6). both contributed to an impaired ability to meet the increased ventilatory demands during physical activity (7). In these conditions, the respiratory system operates outside of the ideal pressure-volume relationship, compromising the force-generating capacity of the inspiratory muscles (7). CRDs pose distinct physiological challenges that impact exercise tolerance and overall respiratory function. In conditions like COPD and pulmonary hypertension (PH), the reduced capacity to generate adequate respiratory muscle force impairs ventilation, thereby limiting exercise capacity (8–10).

Inspiratory muscle training (IMT), defined as a rehabilitative intervention that strengthens the inspiratory muscles through the use of inspiratory threshold loading devices that provide a fixed resistance to inhalation (11), has emerged as a promising intervention, enhancing aerobic capacity by strengthening the respiratory muscles, exercise capacity is improved due to the potential increases thoracic expansion and operating lung volumes reducing dynamic hyperinflation, and to the improved work of breathing of the primary inspiratory muscles (diaphragm), thereby reducing respiratory muscle fatigue, dyspnea, and attenuating the concomitant 'stealing' effect of blood flow from the exercising limb to the respiratory muscles (12–14).

Most studies explaining the potential mechanisms of IMT have been conducted in healthy subjects. These studies have shown that IMT can: (1) decrease the inspiratory muscle motor drive while preserving pressure generation; (15) (2) promote hypertrophy of the diaphragm and increase the proportion of type I fibres and the size of type II fibres in the external intercostal muscles; (16) (3) decrease the rating of perceived dyspnoea or rating of perceived exertion; (17) and (4) improve respiratory muscle endurance (18).

These physiological findings provide a foundation for understanding how IMT can benefit individuals with CRDs, but they also underscore the need for further research to better define its impact across different disease stages and types. However, there have been inconclusive findings regarding the effectiveness of IMT in improving exercise tolerance in patients with CRDs (19). Our objective was to determine the effectiveness of IMT on exercise tolerance, maximum respiratory pressure, lung function, symptoms and quality of life in different CRDs.

## Methods

### Protocol and registration

We performed an overview of systematic reviews (SRs) according to the methodology proposed by the Cochrane Handbook for Systematic Reviews of Interventions (20). This overview was reported using the preferred reporting items for overviews of reviews (PRIOR) statement (21). The review was registered in the International Prospective Register of Systematic Reviews (PROSPERO) with the identifier CRD42022350564.

### Eligibility criteria

We included SRs that focused on interventions with or without meta-analysis, which considered primary studies with a randomised controlled trials (RCTs) design. Network meta-analyses were excluded. The studies included in must have involved adults with CRDs. Furthermore, only SRs that examined IMT interventions, excluding pulmonary rehabilitation, were considered. Regarding comparators, we included SRs where control groups in the primary studies received usual care, placebo, or sham treatments. Lastly, we focused on SRs that addressed the effectiveness of IMT interventions in at least one of the following outcomes: exercise tolerance, pulmonary function, maximum inspiratory or expiratory pressure, dyspnoea, or quality of life.

### Search strategies and data resources

We reviewed five databases: Embase, PubMed/MEDLINE, Web of Science, Cochrane Central Register of Controlled Trials Cochrane Database of Systematic Reviews (CDSR), and Epistemonikos from their inception to March 8, 2025. We imposed no language or publication restrictions. See Supplementary Material S1.

The terms selected were combined using Boolean logical operators (OR, AND, NOT). Moreover, we did a manual search of the references that were included in the selected articles.

### Study selection

Two investigators (RTC-SCT) performed the selection independently. The first step involved reviewing the titles and abstracts of all the references retrieved by the database searches (RTC-SCT) and identifying the SRs that met the inclusion criteria. Next, we selected all articles deemed potentially eligible by at least one of the reviewers. In the second step, we reviewed the full texts, and a decision on inclusion or exclusion was made according to the predefined selection criteria (RTC-SCT). A third reviewer (LVC) solved any disagreement in any step. We excluded studies that did not fulfil the inclusion criteria, and their bibliographic details were listed with the specific reason for exclusion.

### Data extraction and methodological quality assessment

Two authors (RTC-SCT) extracted the data independently and in duplicate, using a standardised protocol and reporting forms. The following information was extracted from each included study: bibliometric characteristics of the publication, general characteristics of the SRs, reported outcome data, quality, or risk of bias of the primary studies included, and certainty of evidence. In addition, the methodological quality of the included SRs was rated in this form.

Two reviewers (RTC-SCT) independently assessed the methodological quality of the SRs included in this overview using “A MeaSurement Tool to Assess systematic Reviews 2” (AMSTAR 2) (22). Disagreements were resolved by consensus or by a third reviewer (LVC). SRs were classified according to the overall confidence in their results as High (no or one non-critical weakness), Moderate (more than one non-critical weakness), Low (one critical flaw with or without non-critical weaknesses), and Critically Low (more than one critical flaw with or without non-critical weaknesses).

### Certainty of the evidence

We used the reported data from one SR chosen for each outcome (“best” SR) to determine the certainty of the evidence using the “Grades of Recommendation, Assessment, Development, and Evaluation” (GRADE) framework (23). Study limitations, inconsistency of results, indirectness of evidence, imprecision, and reporting bias are assessed by the GRADE approach.

### Data analysis

The unit of analysis of this overview was the SRs. Therefore, the primary studies included by each SR were not accessed in case of missing data. Due to the possible existence of redundant SRs, strategies were applied to visualise (24), calculate (25), and manage the overlap (26). In the first instance, a matrix was created with cross-references of the SRs included in this overview with the primary studies included by these SRs. This was done at the outcome level. In addition, from these matrices, the corrected covered area (CCA) (25) were calculated without considering any structural missing data and considering the chronological structural missing data and by primary study design. The Graphical Representation of Overlap for OVErviews (GROOVE) tool was used (27). To select the “best” SR for reporting the effectiveness of each outcome, we prioritise the SR that contains the highest number of primary studies included, rated with a higher rating in their results according to AMSTAR 2.

## Results

### Study selection

**Figure 1:**
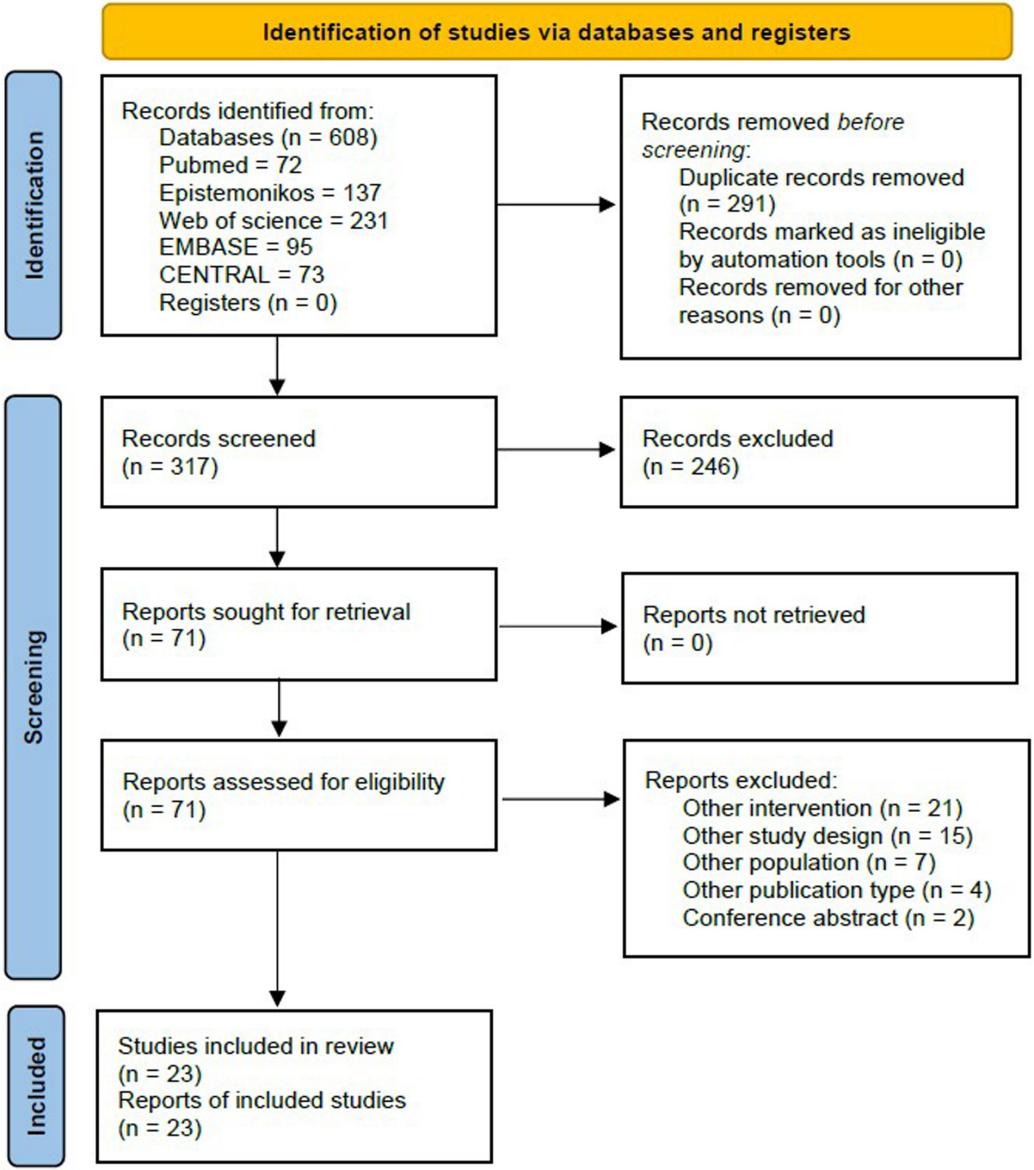
Flowchart of the included studies.

From the 608 identified references, we removed 291 duplicated and screened 317 SRs. We had 71 SRs assessed based on the full text. We excluded 21 SRs for other intervention, 15 for other study design, seven for other population, four for other publication type, and two SRs for being conference abstracts. Finally, 23 SRs were included (Figure 1).

### Characteristics of the included SRs

We included ten SRs in patients with COPD (28–37), five in patients with asthma (17, 38–41), four with obstructive sleep apnoea (OSA) patients (42–45), two with PH (46, 47), one in patients with lung resection (48), and one in patients with bronchiectasis (49). Out of the 23 SRs, 21 were SRs with meta-analysis, and two did not pool outcome data from primary studies (Table 1).

**Table 1:** AMSTAR-2 assessment of each included systematic reviews.

Study	AMSTAR-2 Items																Rating
	1	2	3	4	5	6	7	8	9	10	11	12	13	14	15	16	
Lötters F. et al, (33)	Y	N	Y	PY	N	N	N	N	PY	N	Y	N	N	Y	N	N	Critically Low
Ram FS. et al, (39)	Y	Y	Y	PY	Y	Y	Y	Y	Y	N	Y	N	N	Y	N	Y	Critically Low
Crowe J. et al, (28)	Y	N	Y	PY	Y	Y	N	Y	Y	N	Y	N	N	Y	N	N	Critically Low
Geddes EL. et al, (30)	Y	N	Y	PY	Y	Y	N	Y	Y	N	Y	N	N	N	N	Y	Critically Low
Geddes EL. et al, (31)	Y	N	N	Y	Y	Y	N	Y	PY	N	Y	N	N	Y	N	Y	Critically Low
O'Brien K. et al, (35)	Y	N	Y	N	Y	Y	N	PY	PY	N	Y	N	N	N	N	Y	Critically Low
Shoemaker MJ, et al, (36)	Y	N	Y	PY	N	N	Y	PY	N	N	NA	NA	N	N	NA	N	Critically Low
Gosselink R. et al, (32)	Y	N	Y	PY	Y	N	Y	Y	Y	N	Y	Y	N	N	N	Y	Critically Low
Nakamiti M. et al, (34)	Y	N	Y	PY	N	N	N	PY	Y	N	NA	NA	N	N	NA	N	Critically Low
Silva IS. et al, (40)	Y	Y	Y	Y	Y	Y	Y	Y	Y	N	Y	Y	Y	Y	N	Y	Low Quality
Figueiredo RIN. et al, (29)	Y	Y	Y	PY	Y	Y	N	N	Y	N	Y	N	N	N	N	Y	Critically Low
Martín-Valero R. et al, (49)	Y	Y	Y	PY	Y	Y	Y	PY	Y	N	Y	N	N	N	N	Y	Critically Low
Chen TA. et al, (17)	Y	Y	Y	PY	Y	Y	Y	PY	Y	N	Y	Y	Y	Y	N	Y	Low Quality
Dar JA. et al, (43)	Y	Y	Y	PY	Y	Y	Y	PY	Y	N	Y	Y	Y	Y	N	N	Low Quality
Luo Z. et al, (47)	Y	Y	Y	PY	Y	Y	Y	PY	Y	N	Y	N	N	Y	N	Y	Critically Low
Torres-Castro R. et al, (44)	Y	Y	Y	PY	Y	Y	Y	Y	Y	N	Y	Y	Y	Y	N	Y	Low quality
Wang Q. et al, (41)	N	N	Y	PY	N	Y	N	Y	Y	N	Y	N	N	N	N	N	Critically Low
Yang MX. et al, (48)	Y	Y	Y	PY	Y	N	Y	PY	Y	N	Y	N	N	Y	N	Y	Critically Low
Ammous et al, (37)	Y	Y	Y	Y	Y	Y	Y	Y	Y	Y	Y	PY	Y	Y	Y	Y	High quality
Chen TA. et al, (17)	Y	Y	Y	PY	Y	Y	Y	PY	Y	N	Y	Y	Y	Y	N	Y	Low Quality
Gutierrez-Arias R. et al, (46)	Y	Y	Y	Y	Y	Y	Y	Y	Y	N	Y	Y	Y	Y	Y	Y	High quality
Lista-Paz A. et al, (38)	Y	Y	Y	PY	Y	Y	Y	Y	Y	N	Y	Y	Y	Y	N	Y	Low Quality
Silva de Sousa et al, (45)	Y	PY	Y	PY	Y	Y	N	PY	Y	N	Y	Y	Y	Y	N	Y	Low Quality

**N**, no; **NA**, not aplicable; **PY**, partial yes; **Y**, yes.

### Risk of bias (RoB) assessment

Only two of the SRs was rated as high quality (37, 46), while six were rated as low quality (38, 40, 42–45) and 14 were rated critically low quality (17, 28–36, 39, 41, 47–49). If we analysed by publication years, 90% of 10 articles published before 2020 was critically low quality; indeed, in 13 articles after 2020, only 50% was critically low. The items that the authors failed to answer the most were: (10) Did the review authors report on the funding sources for the studies included in the review? (4.3%), (15) If they performed quantitative synthesis, did the review authors carry out an adequate investigation of publication bias (small study bias) and discuss its likely impact on the results of the review? (8.6%), and (13) Did the review authors account for RoB in individual studies when interpreting/discussing the results of the review? (39.3%) (Table 2). The RoB of each SR reported by the authors is in Supplementary Material S2.

**Table 2:** Outcomes reported by the selected studies.

Author, year	Country	Disease	SR/Studies included	Meta-analysis	MA/Studies included	Physical Capacity	MIP	MEP	Dyspnea	Lung function	QoL
Lotters F. et al, (33)	Belgium/The Netherlands	COPD	15	Yes	15	x	x		x		x
Ram FS. et al, (39)	New Zealand/UK	Asthma	5	Yes	4		x	x		x	
Crowe J. et al. (28)	Canada	COPD	16	Yes	16	x	x			x	x
Geddes EL. et al, (30)	Canada	COPD	19	Yes	15	x	x		x	x	x
Geddes EL. et al, (31)	Canada	COPD	16	Yes	16	x	x		x	x	x
ÓBrien K. et al, (35)	Canada	COPD	18	Yes	6	x	x		x		x
Shoemaker MJ. et al. (36)	USA	COPD	15	No	NA	x	x		x		x
Gosselink R. et al, (32)	Belgium/The Netherlands	COPD	32	Yes	32	x	x		x		x
Nakamiti M. et al, (34)	Brazil	COPD	11	No	NA	x	x		x	x	x
Silva IS. et al. (40)	Brazil	Asthma	5	Yes	4		x	x	x	x	
Figuereido RIN. et al, (29)	Brazil	COPD	48	Yes	48	x	x		x	x	x
Martín-Valero R. et al, (49)	Spain	Bronchiectasis	9	Yes	4	x	x	x		x	x
Chen Y et al, (17)	China	Asthma	6	Yes	6		x		x	x	
Dar JA. et al, (43)	India	OSA	7	Yes	7	x	x			x	
Luo Z. et al, 2022	China	PH	4	Yes	3	x	x		x	x	x
Torres-Castro R. et al, (47)	Chile	OSA	8	Yes	6	x	x				
Wang Q. et al, (41)	China	Asthma	13	Yes	13		x	x		x	
Yang et al, (48)	China	Pulmonary resection	7	Yes	7	x	x	x	x	x	x
Ammous et al, (37)	Tunisia	COPD	37	Yes	32	x	x	x	x	x	x
Chen TA et al, (17)	China	OSA	7	Yes	6		x				
Gutiérrez-Arias R. et al, (46)	Chile	PH	7	Yes	4	x	x	x	x	x	x
Lista-Paz A. et al, (38)	Spain	Asthma	11	Yes	10	x	x	x		x	x
Silva de Sousa et al, (45)	Brazil	OSA	13	Yes	6	x	x			x	

COPD, chronic obstructive pulmonary disease; MEP, maximum expiratory pressure; MIP, maximum inspiratory pressure; OSA, obstructive sleep apnea; PH, pulmonary hypertension; QoL, quality of life.

### Main findings

#### COPD

**Figure 2:**
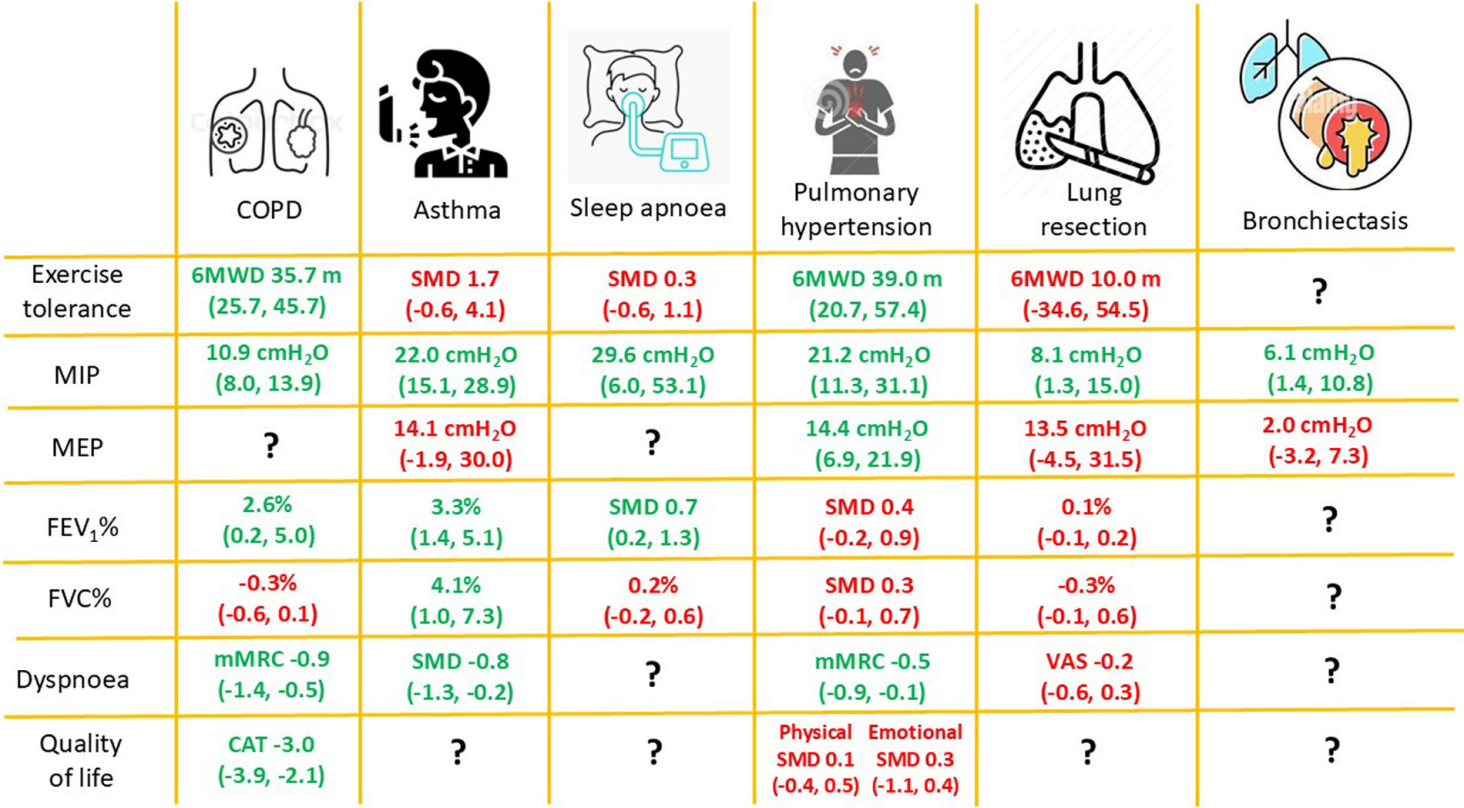
Representation of main findings. 6MWD, six-minute walk distance; COPD, chronic obstructive pulmonary disease; CRQ, chronic respiratory disease questionnaire; FEV_1_, forced expiratory volume in the first second; FVC, forced vital capacity; MEP, maximum expiratory pressure; MIP, maximum inspiratory pressure; mMRC, modified medical research council; SMD, standard mean difference; VAS, visual analogue scale.

We selected ten SRs, eight of them performed meta-analyses. All SRs reported exercise capacity and MIP as outcomes, nine reported dyspnoea and quality of life, seven reported respiratory resistance, and six reported lung function. There was a very high overlap between the SRs in exercise tolerance and MIP, high overlap in forced expiratory volume in the first second (FEV_1_), dyspnoea and quality of life, and moderate overlap in forced vital capacity (FVC) (Table 3). IMT increased exercise tolerance by 35.7 (95% CI 25.7, 45.7) metres in the 6MWT (37), FEV_1_ by 2.6%pred (95% CI 0.2, 5.0), and MIP by 10.9 cmH_2_O (95% CI 8.0, 13.9) (37). Dyspnoea was reduced by 0.6 (95% CI 0.4, 0.8) points in the Borg Scale and 0.9 (95% CI 0.51–1.36) points in modified Medical Research Council (mMRC), and quality of life was improved by 3 (95% CI 2.1, 3.9) points in COPD assessment test (CAT), but the Saint George Respiratory Questionnaire (SGRQ) had not changes (−3.9, 95% CI −8.2, −0.5) (37). The FVC had no changes (−0.3%pred, CI 95% −0.6, 0.1) (29). Maximum expiratory pressure (MEP) was not reported. The certainty of the evidence according to the GRADE methodology was moderate for exercise tolerance, and quality of life (measured with CAT) low for MIP, FEV_1_, and dyspnoea (measured with Borg scale), and very low for FVC, dyspnoea (measured with mMRC), and quality of life (measured with SGRQ), (Table 4) (Figure 2).

**Table 3:** Overlap level by disease and outcome.

Outcome	COPD			Asthma			OSA			PH			Lung resection		
	Number of SRs	CCA		Number of SRs	CCA		Number of SRs	CCA		Number of SRs	CCA		Number of SRs	CCA	
		%	Interpretation		%	Interpretation		%	Interpretation		%	Interpretation		%	Interpretation
Exercise tolerance	9	20.1	Very high overlap	1	NA	NA	3	100	Very high overlap	2	57.1	Very high overlap	1	NA	NA
FEV_1_	4	14.7	High overlap	5	28.1	Very high overlap	2	100	Very high overlap	2	60.0	Very high overlap	1	NA	NA
FVC	3	8.3	Moderate overlap	5	28.1	Very high overlap	2	100	Very high overlap	2	60.0	Very high overlap	1	NA	NA
MIP	9	23.1	Very high overlap	5	37.5	Very high overlap	4	61.9	Very high overlap	2	50.0	Very high overlap	1	NA	NA
MEP	0	NA	NA	4	22.2	Very high overlap	0	NA	NA	2	50.0	Very high overlap	1	NA	NA
Dyspnoea	9	14.2	High overlap	2	0	No overlap	0	NA	NA	2	50.0	Very high overlap	1	NA	NA
QoL	9	11.1	High overlap	0	NA	NA	0	NA	NA	2	60.0	Very high overlap	2	0%	NA

CCA, corrected covered area; COPD, chronic obstructive pulmonary disease; FEV_1_, forced expiratory volume in the first second; FVC, forced vital capacity; MEP, mMaximum expiratory pressure; MIP, maximum inspiratory pressure; NA, not applicable; OSA, obstructive sleep apnea; PH, pulmonary hypertension; QoL, auality of life.

**Table 4:** Summary of findings.

Disease	Outcomes	Corresponding risk	Selected SR	Certainty
		IMT (CI 95%)	(studies)	
COPD	Exercise tolerance—metres in 6MWT	MD 35.7 higher	Ammous et al.	⊕⊕⊕⊝
		(25.7–45.7 higher)	(16 studies)	Moderate
	Maximum inspiratory pressure—cmH_2_O	MD 10.9 higher	Figuereido et al.	⊕⊕⊝⊝
		(8.0–13.9 higher)	(36 studies)	Low
	FEV_1_—%pred	MD 2.6 higher	Ammous et al.	⊕⊕⊝⊝
		(0.2–5.0 higher)	(10 studies)	Low
	FVC—%pred	MD 0.3 lower	Figuereido et al.	⊕⊝⊝⊝
		(0.6 lower–0.1 higher)	(10 studies)	Very low
	Dyspnoea—mMRC	MD 0.94 lower	Ammous et al.	⊕⊝⊝⊝
		(0.51–1.36 lower)	(6 studies)	Very Low
	Dyspnoea—Borg scale	MD 0.59 lower	Ammous et al.	⊕⊕⊝⊝
		(0.43–0.76 lower)	(4 studies)	Low
	Quality of life—SGRQ	MD 3.9 lower	Ammous et al.	⊕⊝⊝⊝
		(8.2 lower–0.5 higher)	(6 studies)	Very Low
	Quality of life—CAT	MD 3.0 lower	Ammous et al.	⊕⊕⊕⊝
		(2.1–3.9 lower)	(2 studies)	Moderate
Asthma	Exercise tolerance—SMD	SMD 1.7 higher	Lista-Paz et al.	⊕⊕⊝⊝
		(0.6 lower–4.1 higher)	(3 studies)	Low
	Maximum inspiratory pressure—cmH_2_O	MD 22.0 higher	Lista-Paz et al.	⊕⊕⊕⊝
		(15.1–28.9 higher)	(9 studies)	Moderate
	Maximum expiratory pressure—cmH_2_O	MD 14.1 higher	Wang et al.	⊕⊝⊝⊝
		(1.9 lower–30.0 higher)	(4 studies)	Very low
	FEV_1_—%pred	MD 3.3 higher	Wang et al.	⊕⊕⊝⊝
		(1.4–5.1 higher)	(9 studies)	Low
	FVC—%pred	MD 4.1 higher	Wang et al.	⊕⊕⊝⊝
		(1.0–7.3 higher)	(8 studies)	Low
	Dyspnoea—SMD	SMD 0.8 lower	Chen et al.	⊕⊝⊝⊝
		(0.2–1.3 lower)	(2 studies)	Very low
Lung resection	Exercise tolerance—metres in 6MWT	MD 10.0 higher	Yang et al.	⊕⊝⊝⊝
		(−34.6 lower–54.5 higher)	(3 studies)	Very low
	Maximum inspiratory pressure—cmH_2_O	MD 8.1 higher	Yang et al.	⊕⊕⊝⊝
		(1.3–14.9 higher)	(5 studies)	Low
	Maximum expiratory pressure—cmH_2_O	MD 13.5 higher	Yang et al.	⊕⊕⊝⊝
		(4.5 lower–31.5 higher)	(4 studies)	Low
	FEV_1_—%pred	MD 0.1 higher	Yang et al.	⊕⊕⊝⊝
		(0.1 lower–0.2 higher)	(3 studies)	Low
	FVC—%pred	MD 0.3 higher	Yang et al.	⊕⊕⊝⊝
		(0.1 lower–0.6 higher)	(2 studies)	Low
	Dyspnoea—VAS (points)	MD 0.2 lower	Yang et al.	⊕⊝⊝⊝
		(0.6 lower–0.3 higher)	(2 studies)	Very low
Pulmonary hypertension	Exercise tolerance—metres in 6MWT	MD 39.0 higher	Gutiérrez-Arias et al.	⊕⊝⊝⊝
		(20.7–57.4 higher)	(4 studies)	Very low
	Maximum inspiratory pressure—cmH_2_O	MD 21.2 higher	Gutiérrez-Arias et al.	⊕⊝⊝⊝
		(11.3–31.1 higher)	(4 studies)	Very low
	Maximum expiratory pressure—cmH_2_O	MD 14.4 higher	Gutiérrez-Arias et al.	⊕⊕⊝⊝
		(6.9–21.9 higher)	(4 studies)	Low
	FEV_1_—%pred	MD 0.4 higher	Gutiérrez-Arias et al.	⊕⊝⊝⊝
		(0.2 lower–0.9 higher)	(4 studies)	Very low
	FVC—%pred	MD 0.3 higher	Gutiérrez-Arias et al.	⊕⊝⊝⊝
		(0.1 lower–0.7 higher)	(4 studies)	Very low
	Dyspnoea—points in mMRC	MD 0.5 lower	Gutiérrez-Arias et al.	⊕⊝⊝⊝
		(0.1–0.9 lower)	(2 studies)	Very low
	Quality of life—Physical	SMD 0.1 higher	Gutiérrez-Arias et al.	⊕⊝⊝⊝
		(0.4 lower–0.5 higher)	(3 studies)	Very low
	Quality of life—Emotional	SMD 0.3 lower	Gutiérrez-Arias et al.	⊕⊝⊝⊝
		(1.1 lower–0.4 higher)	(3 studies)	Very low
Sleep apnoea	Exercise tolerance—SMD	SMD 0.3 higher	Torres-Castro et al.	⊕⊕⊝⊝
		(0.6 lower–1.1 higher)	(3 studies)	Low
	Maximum inspiratory pressure—cmH_2_O	MD 29.6 higher	Torres-Castro et al.	⊕⊕⊕⊝
		(6.0–53.1 higher)	(6 studies)	Moderate
	FEV_1_—SMD	SMD 0.7 higher	Dar et al.	⊕⊕⊝⊝
		(0.2–1.3 higher)	(3 studies)	Low
	FVC—%pred	MD 0.2 higher	Chen et al.	⊕⊕⊝⊝
		(0.2 lower–0.6 higher)	(2 studies)	Low
Bronchiectasis	Maximum inspiratory pressure—cmH_2_O	MD 6.1 higher	Martin-Valero et al.	⊕⊕⊝⊝
		(1.4–10.8 higher)	(4 studies)	Low
	Maximum expiratory pressure—cmH_2_O	MD 2.0 higher	Martin-Valero et al.	⊕⊝⊝⊝
		(3.2 lower–7.3 higher)	(3 studies)	Very low

NOTE: GRADE Working Group grades of evidence.

⊕⊕⊕⊕ High certainty: We are very confident that the true effect lies close to that of the estimate of the effect.

⊕⊕⊕⊝ Moderate certainty: We are moderately confident in the effect estimate: The true effect is likely to be close to the estimate of the effect, but there is a possibility that it is substantially different.

⊕⊕⊝⊝ Low certainty: Our confidence in the effect estimate is limited: The true effect may be substantially different from the estimate of the effect.

⊕⊝⊝⊝ Very low certainty: We have very little confidence in the effect estimate: The true effect is likely to be substantially different from the estimate of effect.

Abbreviations 6MWT, six-minute walk test; CAT, COPD assessment test; CI, confidence interval; COPD, chronic obstructive pulmonary disease; CRQ, chronic respiratory questionnaire; FEV_1_, forced expiratory volume in the first second; FVC, forced vital capacity; MD, mean difference; mMRC, modified medical research council; SGRQ, saint george respiratory questionnaire; SMD, standard mean difference; VAS, visual analogue scale.

#### Asthma

We selected five SRs that included patients diagnosed with asthma. All of them performed meta-analyses. All SRs reported MIP and lung function as outcomes, three reported maximum expiratory pressure (MEP), two reported dyspnoea, and one reported exercise tolerance. There was a very high overlap between SRs in FEV_1_, FVC, MIP, and MEP (Table 3). IMT increased FEV_1_ by 3.3%pred (95% CI 1.4, 5.1) (41), FVC by 4.1%pred (95% CI 1.0, 7.3) (41), MIP by 22.0 cmH_2_O (95% CI 15.1, 28.9) (38). Dyspnoea was reduced (SMD −0.8, 95% CI −1.3, −0.2) (17). No changes were found in exercise tolerance (SMD 1.7, 95% CI −0.6, 4.1) (38) and MEP (14.1 cmH_2_O, 95% CI −1.9, 30.0) (41). The quality of life was not reported. The certainty of the evidence, according to the GRADE methodology, was moderate for MIP, low for exercise tolerance, FEV_1_ and FVC, and very low for MEP and dyspnoea (Table 4; Figure 2).

Other outcomes reported, but not pooled, were: (1) use of rescue medication, of which, three of four SRs found a significant decrease in β2-agonist consumption un the IMT group; (38) (2) asthma-related symptoms and asthma control with different results (38).

#### Obstructive sleep apnoea (OSA)

We selected four SRs that included patients with OSA (42–45). All were performed meta-analyses. There was a very high overlap between SRs in exercise tolerance and MIP (Table 3). IMT increased MIP by 29.6 cmH_2_O (95% CI 6.0, 53.1) (44), and FEV_1_ by 0.7 SMD (95% CI 0.2, 1.3) (43). The exercise tolerance did not improve (SMD 0.3, 95% CI −0.6, 1.1) (44). No changes were found in FVC (0.2%pred, 95% CI −0.2, 0.6) (42). The MEP, dyspnoea and quality of life were not reported. The certainty of the evidence according to the GRADE methodology was moderate for MIP, and low for exercise tolerance, FEV_1_ and FVC (Table 4; Figure 2).

Other outcomes reported were: (1) Apnoea/hypopnea index, with all SRs found no changes; (42–45) (2) Sleepiness and sleep quality with an improvement in all SRs; (42–45) and (3) Blood pressure with two SRs that on analysis was found to show an improvement in systolic (42, 45) and diastolic blood pressures (42).

#### Pulmonary hypertension

We selected two SRs that included patients with PH (46, 47). Both performed meta-analyses. There was a very high overlap between SRs in all analysed outcomes (Table 3). IMT increased exercise tolerance by 39.0 metres (95% CI 20.7, 57.4) in the 6MWT, MIP by 21.2 cmH_2_O (95% CI 11.3, 31.1), MEP by 14.4 cmH_2_O (95% CI 6.9, 21.9) (46). Dyspnoea was reduced by 0.5 (95% CI 0.1, 0.9) points in the mMRC scale (46). No changes were found in FEV_1_ (SMD 0.4, 95% CI −0.2, 0.9), FVC (SMD 0.3, 95% CI −0.1, 0.7), and quality of life in physical (SMD 0.1, 95% CI −0.4, 0.5) and emotional dimension (SMD 0.3, 95% CI −1.1, 0.4) (46). The certainty of the evidence, according to the GRADE methodology, was low for MIP, and very low for all other outcomes (Table 4; Figure 2).

Another outcome reported was physical activity. One individual study selected by one SR found that the IMT group increased by 195.2 MET/min/day (95% CI 102.9, 287.6) compared to the sham IMT group (50). In addition, it reported that the sham IMT group increased by 403.8 steps per day (95% CI −1,241.5, 2,049.1) compared to the IMT group (50).

#### Lung resection

We selected one SR that included patients undergoing LR. This SR reported all analysed parameters including studies before and after surgery. IMT increased MIP by 8.1 cmH_2_O (95% CI 1.3, 15.0) (48). Dyspnoea was reduced by SMD −0.8 (95% CI −1.3, −0.2) points (48). No changes were found in exercise tolerance (10.0 m by 6MWT, 95% CI −34.6, 54.5), FEV_1_ (0.1%pred, 95% CI −0.1, 0.2), FVC (−0.3%pred, 95% CI −0.1, 0.6) (48), MEP (13.5, 95% CI −4.5, 31.5), and dyspnoea (VAS −0.2, 95% CI −0.6, 0.3) (41). The quality of life was not reported. The certainty of the evidence, according to the GRADE methodology, was low for lung function, MIP and MEP, and very low for exercise tolerance and dyspnoea (Table 4; Figure 2).

Other outcomes reported were: (1) pain with no significant differences; (48) and (2) physical activity with higher physical activity in the IMT group (48).

#### Bronchiectasis

Only one study included patients with bronchiectasis. This study reported only MIP and MEP. IMT increased MIP by 6.1 cmH_2_O (95% CI 1.4, 10.8) (49). No changes were found in MEP (2.1, 95% CI −3.2, 7.3) (49). Exercise tolerance, lung function, dyspnoea and quality of life were not reported. The certainty of the evidence, according to the GRADE methodology, was low for MIP and very low for MEP (Table 4; Figure 2).

## Discussion

IMT showed beneficial effects on several clinically relevant outcomes across different CRDs. Improvements MIP were observed in asthma, OSA, COPD, PH, lung resection, and bronchiectasis. Exercise tolerance increased in COPD and PH, and lung function improved in asthma and OSA. Additionally, reductions in dyspnoea and improvements in quality of life were noted, particularly in COPD patients. These findings highlight the effectiveness of IMT in CRDs.

Exercise tolerance was increased in COPD and PH, close to the minimal clinically important difference (MCID) reported for CRD (51). Our results show that this improvement may be clinically significant. A possible explanation for this improvement is provided by Welch et al., who hypothesized that IMT enhances the aerobic capacity of the respiratory muscles, thereby reducing their demand for blood flow during exercise (12). As a result, a greater proportion of cardiac output could be redirected to the locomotor muscles, potentially delaying peripheral fatigue and improving exercise performance. This mechanism is supported by findings showing that unloading the inspiratory muscles can reduce diaphragm and locomotor muscle fatigue, and prolong exercise duration, particularly during high-intensity efforts (12). In patients with PH, it is proposed that improving MIP, reducing the severity of exertional dyspnea, and enhancing quadriceps muscle strength through IMT play a crucial role in enhancing exercise performance (52). Asthma and OSA did not improve exercise tolerance, coinciding that these were the meta-analyses with fewer studies (three each) so they should be analysed with caution.

Improved lung function was observed in asthma and OSA, specifically FEV_1_. A possible explanation of this small increase is due to lung function parameters are force-dependent and reflect lung capacity and mainly affected by respiratory muscle strength, airway resistance and lung compliance (41). It is possible that, as a result of IMT, the respiratory muscles were able to perform more work, which resulted in an improvement in respiratory capacity and an increase in thoracic expansion and, therefore, had a role in increasing lung volumes (13). Although in the case of COPD, FEV_1_ increased significantly, the change is only 2.6%, this result is statistically significant, however, its clinical impact is debatable (and likely low) and it is smaller even than the margin of error of this measurement (53).

MIP was increased in all CRDs analysed. However, in asthma, lung resection and bronchiectasis the change was lower than the MCID established in COPD (54). Unfortunately, to date there is no established MCID for these populations (38). Regarding the changes in MEP, with the exception of patients with OSA, we did not find significant differences in the other CRDs, probably due to the specificity of the IMT that complies with the physiological principles of training (55).

Evidence of dyspnoea diminution was found only in COPD, asthma and PH. A possible explanation is due that IMT can have a possible effect on dynamic hyperinflation, commonly observed in obstructive diseases, allowing the diaphragm to work with a better force-length relationship and allowing for the generation of a given pressure with less respiratory motor drive (14). Individuals with different levels of lung obstruction have less dyspnoea when they have higher MIP (56). On the other hand, the literature has shown that inspiratory muscle fatigue causes the metaboreflex, resulting in vasoconstriction of the blood vessels in the peripheral muscles, which leads to a decrease in respiratory performance (57). IMT might minimise the effects of the activation of the inspiratory muscle metaboreflex.

In COPD patients, the reduction in diaphragmatic electromyographic activity (EMG_di_) and the EMG_di_/EMG_dimax_ ratio after IMT suggests an improvement in diaphragmatic efficiency, leading to less muscular effort during breathing (58). This could translate into a decrease in the perception of dyspnea, even during intense exercise, as the body no longer needs to recruit as much effort from the diaphragm to maintain minute ventilation (VE) during physical activity (58).

In PH, the primary symptom is progressively worsening dyspnea, often occurring with minimal effort (59). The heightened ventilation demand, coupled with reduced respiratory muscle function, leads to an increased neural respiratory drive, thereby intensifying the sensation of breathlessness (59). Additionally, patients with PH have been found to experience respiratory muscle weakness and atrophy, which contribute to the sensation of dyspnea and exercise-induced fatigue (8).

Evidence of quality of life improvement was found for COPD. There is a close relationship between dyspnoea and quality of life. It has been reported that patients who perceive severe dyspnoea have a worse quality of life (60). However, the CRQ had a change lower than the MCID (61), so it must be analysed with caution since it does not represent a clinically significant change. In PH, however, it did not improve, although it was only analysed in three studies (46), so future studies in this disease should explore the change in this outcome.

We found a very high overlap in the studies included in the SRs for MIP and lung function in asthma, OSA and PH, and dyspnoea and quality of life in COPD, however, there was no overlap in the studies related to dyspnea in asthma. The articles selected in this overview were aimed at answering the same research question, which is why it is striking that there was no overlap in some outcomes. On the other hand, we found many SRs aimed at answering the same question in COPD. This should make us reflect on the meaning of carrying out the same SRs with little time between them.

Most of the SRs included in the study were of “low” quality or “critically low”. AMSTAR 2 has previously been used in several fields, and many publications have reported a substantial number of SRs with low or critically low quality (62). AMSTAR 2, a critical appraisal tool, was introduced in 2017, at a time when most SRs were utilizing different guidelines for their methodologies (62). Consequently, until 2020, fewer than 200 publications in PubMed implemented AMSTAR 2 in their study approaches (62). This lack of adoption and adherence to AMSTAR 2 guidelines may explain certain instances of non-compliance in the conduct and reporting of research studies (62). For this reason, the risk of bias of the results should be carefully assessed along with the risk of bias assessment of each SR individually. Given the high prevalence of low and critically low-quality SRs, the implications of these findings should be carefully considered. The lack of adherence to established guidelines, such as AMSTAR 2, likely contributes to the inconsistencies and limitations observed in the SRs, which must be taken into account when interpreting the results.

There is significant uncertainty regarding the evidence on IMT, highlighting the need for better-designed studies with adequate statistical power rather than continuing to accumulate inconclusive evidence. This overview provides a snapshot of the current landscape and suggests that future studies should focus on less explored CRDs such as interstitial lung diseases or bronchiectasis, to avoid extrapolating findings from COPD. Additionally, the need for new SRs, should be carefully assessed, as many reliable ones already exist; improving the evidence base will require high-quality primary studies (63). Our GRADE-based analysis also revealed discrepancies among SRs, suggesting inconsistent application of criteria when determining the certainty of evidence.

### Strengths and limitations

The great strength of this overview is that it allows analysing a broad spectrum of chronic diseases with relevant outcomes, including patient reported outcome measures (PROMs), and, in this way, clinicians can quickly see if the intervention is helpful for the population they treat. On the other hand, we only consider SRs with meta-analyses of RCTs, which strengthens the conclusions. Nevertheless, since we did not perform a meta-analysis, some RCTs could have been left out of the results and findings if not in the “best” SR.

Among the limitations we found is the lack of SRs of other CRDs, such as interstitial diseases and cystic fibrosis (excluded for mixing children and adults). Another important limitation not addressed by this review is the variety of devices and different training protocols used. This issue has been reported and is probably one of the aspects that determines the heterogeneity of the results of the included studies and the discussion of the effectiveness of IMT in some pathologies. The variation in training protocols, devices, duration, and intensities may influence the results and limit the ability to draw firm conclusions.

## Conclusion

IMT improved several clinically relevant outcomes, including MIP, exercise capacity, and dyspnoea in different CRDs. However, the limited evidence for certain outcomes and populations highlights the need for further high-quality studies. Additionally, the interpretation of these results should be approached with caution, given the variable quality of the SRs included in this study.
