# Supplementary material for: Effectiveness of inspiratory muscle training in patients with a chronic respiratory disease: an overview of systematic reviews
Source: Front Sports Act Living. 2025 May 21;7:1549652. doi: 10.3389/fspor.2025.1549652 (PMC12133981; doi:10.3389/fspor.2025.1549652)
Supplement: Supplementary file 1 [file Table1.docx]

**PubMed**

Search date: 08/03/2025; Restricted to studies published until 08/03/2025

| # | SEARCH TERMS | N° OF RESULTS |
| --- | --- | --- |
| #1 | (Chronic obstructive pulmonary disease[Title/Abstract] OR COPD[Title/Abstract] OR Asthma[Title/Abstract] OR Bronchiectasis[Title/Abstract] OR Pulmonary hypertension[Title/Abstract] OR Pulmonary arterial hypertension[Title/Abstract] OR Interstitial lung diseases[Title/Abstract] OR Idiopathic pulmonary fibrosis[Title/Abstract] OR Cystic fibrosis[Title/Abstract] OR Sarcoidosis[Title/Abstract] OR Lung transplant[Title/Abstract] OR obstructive sleep apnea[Title/Abstract] OR lung cancer[Title/Abstract] OR lung volume reduction surgery[Title/Abstract] OR endobronchial valve[Title/Abstract]) | 677,584 |
| #2 | (inspiratory muscle training[Title/Abstract] OR Respiratory muscle training[Title/Abstract] OR IMT[Title/Abstract] OR breathing exercises[Title/Abstract]) | 12,609 |
| #3 | (maximum inspiratory pressure[Title/Abstract] OR maximum expiratory pressure[Title/Abstract] OR Cardiopulmonary Exercise Test[Title/Abstract] OR Exercise Tolerance[Title/Abstract] OR Exercise Test[Title/Abstract] OR Exercise Capacity[Title/Abstract] OR Cardiorespiratory Fitness[Title/Abstract] OR Oxygen Consumption[Title/Abstract] OR Aerobic Capacity[Title/Abstract] OR Six-minute walk test[Title/Abstract] OR 6MWT[Title/Abstract] OR Shuttle walking test[Title/Abstract] OR SWT[Title/Abstract] OR Distance walked[Title/Abstract] OR Dyspnea[Title/Abstract] OR fatigue[Title/Abstract] OR Quality of life[Title/Abstract] OR physical activity[Title/Abstract] OR survival[Title/Abstract]) | 2,083,287 |
| #4 | #1 AND #2 AND #3 | 496 |
| #5 | #4 / filter systematic review | 72 |

**Epistemonikos**

Search date: 08/03/2025; Restricted to studies published until 08/03/2025

| # | SEARCH TERMS | N° OF RESULTS |
| --- | --- | --- |
| #1 | ((Chronic obstructive pulmonary disease) OR (COPD) OR (Asthma) OR (Bronchiectasis) OR (Pulmonary hypertension) OR (Pulmonary arterial hypertension) OR (Interstitial lung diseases) OR (Idiopathic pulmonary fibrosis) OR (Cystic fibrosis) OR (Sarcoidosis) OR (Lung transplant) OR (obstructive sleep apnea) OR (lung cancer) OR (lung volume reduction surgery) OR (endobronchial valve)) | 188,286 |
| #2 | ((inspiratory muscle training) OR (Respiratory muscle training) OR (IMT) OR (breathing exercises)) | 6,751 |
| #3 | ((maximum inspiratory pressure) OR (maximum expiratory pressure) OR (Cardiopulmonary Exercise Test) OR (Exercise Tolerance) OR (Exercise Test) OR (Exercise Capacity) OR (Cardiorespiratory Fitness) OR (Oxygen Consumption) OR (Aerobic Capacity) OR (Six-minute walk test) OR (6MWT) OR (Shuttle walking test) OR (SWT) OR (Distance walked) OR (Dyspnea) OR (fatigue) OR (Quality of life) OR (physical activity) OR (survival)) | 671,304 |
| #4 | #1 AND #2 AND #3 | 877 |
| #5 | #4 / filter systematic review | 137 |

**Web of Science**

Search date: 08/03/2025; Restricted to studies published until 08/03/2025

| # | SEARCH TERMS | N° OF RESULTS |
| --- | --- | --- |
| #1 | (Chronic obstructive pulmonary disease OR COPD OR Asthma OR Bronchiectasis OR Pulmonary hypertension OR Pulmonary arterial hypertension OR Interstitial lung diseases OR Idiopathic pulmonary fibrosis OR Cystic fibrosis OR Sarcoidosis OR Lung transplant OR obstructive sleep apnea OR lung cancer OR lung volume reduction surgery OR endobronchial valve) | 1,095,342 |
| #2 | (inspiratory muscle training OR Respiratory muscle training OR IMT OR breathing exercises) | 27,119 |
| #3 | (maximum inspiratory pressure OR maximum expiratory pressure OR Cardiopulmonary Exercise Test OR Exercise Tolerance OR Exercise Test OR Exercise Capacity OR Cardiorespiratory Fitness OR Oxygen Consumption OR Aerobic Capacity OR Six-minute walk test OR 6MWT OR Shuttle walking test OR SWT OR Distance walked OR Dyspnea OR fatigue OR Quality of life OR physical activity OR survival) | 3,462,623 |
| #4 | #1 AND #2 AND #3 | 2,912 |
| #5 | #4 / filter systematic review | 231 |

**EMBASE**

Search date: 08/03/2025; Restricted to studies published until 08/03/2025

| # | SEARCH TERMS | N° OF RESULTS |
| --- | --- | --- |
| #1 | 'chronic obstructive pulmonary disease':ab,ti OR copd:ab,ti OR asthma:ab,ti OR bronchiectasis:ab,ti OR 'pulmonary hypertension':ab,ti OR 'pulmonary arterial hypertension':ab,ti OR 'interstitial lung diseases':ab,ti OR 'idiopathic pulmonary fibrosis':ab,ti OR 'cystic fibrosis':ab,ti OR sarcoidosis:ab,ti OR 'lung transplant':ab,ti OR 'obstructive sleep apnea':ab,ti OR 'lung cancer':ab,ti OR 'lung volume reduction surgery':ab,ti OR 'endobronchial valve':ab,ti | 1,206,045 |
| #2 | 'inspiratory muscle training':ab,ti OR 'respiratory muscle training':ab,ti OR imt:ab,ti OR 'breathing exercises':ab,ti | 24,432 |
| #3 | 'maximum inspiratory pressure':ab,ti OR 'maximum expiratory pressure':ab,ti OR 'cardiopulmonary exercise test':ab,ti OR 'exercise tolerance':ab,ti OR 'exercise test':ab,ti OR 'exercise capacity':ab,ti OR 'cardiorespiratory fitness':ab,ti OR 'oxygen consumption':ab,ti OR 'aerobic capacity':ab,ti OR 'six-minute walk test':ab,ti OR 6mwt:ab,ti OR 'shuttle walking test':ab,ti OR swt:ab,ti OR 'distance walked':ab,ti OR dyspnea:ab,ti OR fatigue:ab,ti OR 'quality of life':ab,ti OR 'physical activity':ab,ti OR survival:ab,ti | 3,500,273 |
| #4 | #1 AND #2 AND #3 | 920 |
| #5 | #4 / filter systematic review | 95 |

**CENTRAL**

Search date: 08/03/2025; Restricted to studies published until 08/03/2025

| # | SEARCH TERMS | N° OF RESULTS |
| --- | --- | --- |
| #1 | (Chronic obstructive pulmonary disease OR COPD OR Asthma OR Bronchiectasis OR Pulmonary hypertension OR Pulmonary arterial hypertension OR Interstitial lung diseases OR Idiopathic pulmonary fibrosis OR Cystic fibrosis OR Sarcoidosis OR Lung transplant OR obstructive sleep apnea OR lung cancer OR lung volume reduction surgery OR endobronchial valve) | 114,394 |
| #2 | (inspiratory muscle training OR Respiratory muscle training OR IMT OR breathing exercises) | 13,578 |
| #3 | (maximum inspiratory pressure OR maximum expiratory pressure OR Cardiopulmonary Exercise Test OR Exercise Tolerance OR Exercise Test OR Exercise Capacity OR Cardiorespiratory Fitness OR Oxygen Consumption OR Aerobic Capacity OR Six-minute walk test OR 6MWT OR Shuttle walking test OR SWT OR Distance walked OR Dyspnea OR fatigue OR Quality of life OR physical activity OR survival) | 453,998 |
| #4 | #1 AND #2 AND #3 | 2,986 |
| #5 | #4 / filter systematic review | 73 |
