# Supplementary material for: Effectiveness of inspiratory muscle training in patients with a chronic respiratory disease: an overview of systematic reviews
Source: Front Sports Act Living. 2025 May 21;7:1549652. doi: 10.3389/fspor.2025.1549652 (PMC12133981; doi:10.3389/fspor.2025.1549652)
Supplement: Supplementary file 2 [file Table2.docx]

Supplementary material 2. Risk of bias reported by authors

| **Study** | **Assessment** | **Results** |
| --- | --- | --- |
| Lötters F. et al. 2002 | Framework for methodological quality (Smith et al. 1992) | The methodological quality score varied from 12–31 (median 24) of the maximal feasible score of 40 points. The main methodological shortcomings were lack of an adequate description of randomisation procedure, no validity and reliability data of the used outcome measures mentioned, and absence of (double) blinding procedures. |
| Ram FS. et al. 2003 | Jadad quality assessment scale (Jadad et al. 1996) | Four of the included trials were appropriately randomised and double-blinded. One study was reported as randomised and single-blinded. Unfortunately, no one of the included studies adequately described the method of allocation concealment used and all were graded as 'B'. However, all trials (except one, McConnell 1998) scored greater than three on the Jadad methodological quality scale indicating that majority of the included trials were of good methodological quality. |
| Crowe J. et al. 2005 | Jadad quality assessment scale (Jadad et al. 1996) | In 11 studies an intention-to-treat analysis was used in which subjects were analyzed in the groups to which they were assigned. The remaining five studies reported a per-protocol analysis, in which subjects who were not compliant with the intervention were excluded from the analysis. There were no withdrawals in four studies. Withdrawal rates in the other studies ranged up to 59%. The two most common reasons for withdrawals were health reasons (acute exacerbations of COPD and other conditions) and lack of interest or other social reasons. |
| Geddes EL. et al. 2005 | Jadad quality assessment scale (Jadad et al. 1996) | All 16 included studies were described as randomised but only three described the randomisation process. Ten of the 16 studies were described as double-blinded in which both the participants and outcome assessors were blind to the intervention and allocation of participants within groups. Fourteen of the 16 included studies reported on participants who withdrew from the study. Five of these 14 studies reported no withdrawals. Withdrawal rates in the remaining nine studies ranged from 5.7 to 51.1%. Thirteen of the 16 included studies reported that comparison groups were similar at baseline. Two studies did not report on group similarity at baseline and one study had older participants with lower arterial oxygen in the sham group. Intention-to-treat analysis was performed in the three studies that reported no withdrawals and was inferred in nine studies because group participants appeared to be analyzed based on the groups to which they were originally randomized. In the remaining four studies, a per-protocol analysis was conducted whereby participants who were non-adherent to the intervention were excluded from the analysis. |
| Geddes EL. et al. 2008 | Jadad quality assessment scale (Jadad et al. 1996) | All 16 included studies were described as randomised but only three described the randomisation process: either a random number table or a computer-generated random number sequence stratified for sex and severity of airflow obstruction. Ten of the 16 studies were described as double-blinded in which both the participants and outcome assessors were blind to the intervention and allocation of participants within groups. One study was assumed to be double-blinded since the assessors were blinded to the intervention and the allocation of participants within groups, and the participants were unaware of the intervention they received due to the use of sham IMT. Single-blinding occurred in the remaining five studies in which participants were unaware of the intervention they received due to the use of sham IMT. Fourteen of the 16 included studies reported on participants who withdrew from the study. Five of these 14 studies reported no withdrawals. Withdrawal rates in the remaining nine studies ranged from 5.7 to 51.1%. Reasons for withdrawal included: respiratory problems or exacerbations, intercurrent illness, family or transportation issues death, or other reasons, including lack of interest in the program. Thirteen of the 16 included studies reported that comparison groups were similar at baseline. Two studies did not report on group similarity at baseline and one study had older participants with lower arterial oxygen in the sham group. Intention-to-treat analysis was performed in the three studies that reported no withdrawals and was inferred in nine studies because group participants appeared to be analyzed based on the groups to which they were originally randomized. In the remaining four studies, a per-protocol analysis was conducted whereby participants who were non-adherent to the intervention were excluded from the analysis. |
| O'Brien K. et al. 2008 | Jadad quality assessment scale (Jadad et al. 1996) | The authors assumed that an intention-to-treat analysis was performed in 13 of the studies whereby participants at study completion were analysed on the basis of the groups to which they were originally randomized. The remaining five studies reported a per-protocol analysis, in which participants who were non-adherent with the intervention were excluded from the analysis. Four studies reported no withdrawals of participants whereas withdrawal rates in the other studies ranged from 7% to 59% (Table 2). The two most common reasons for withdrawal included health reasons (acute exacerbations of COPD and other conditions), and lack of interest, motivation, and other social reasons that resulted in incompletion of the study. |
| Shoemaker MJ. et al. 2009 | Medlicott and Harris score (Medlicott et al. 2006) | Medlicott and Harris scores of methodological rigour ranged from 40% to 90%. Six articles were rated “strong” eight as “moderate”, and one as “weak”. |
| Gosselink R. et al. 2011 | Framework for methodological quality modified (Smith et al. 1992) | The methodological quality score varied from 30–83% (median 59%) of the maximum score and did not significantly relate to the effect size observed in the studies. |
| Nakamiti et al. 2011 | PEDro scale | Four studies did not obtain the minimum score on the PEDro scale (<5 points). 11 studies were considered to be of moderate-high quality. Four studies scored 5 points, one study scored 6 points, five studies scored 7 points and one study scored 8 points on the PEDro scale. |
| Silva IS. et al. 2013 | Cochrane Collaboration Risk of Bias tool | There were substantial differences between the studies, including the training protocol, duration of training sessions (10 to 30 minutes) and duration of the intervention (over 3 to 25 weeks). The methodological quality of the studies included in this update was difficult to accurately ascertain. Study samples were small and the risk of bias was mostly unclear, due to inadequate reporting. Overall the quality of the evidence included in the review was very low. |
| Figueiredo RIN. et al. 2020 | Cochrane Collaboration Risk of Bias tool | Of the studies included in this systematic review, 15.2% reported allocation concealment, 17.4% reported blinding of therapists, and 30.4% used the principle of intention to treat for statistical analyses, characterizing a high risk of bias for these items. Still, 34.8% used patient blinding, and 39.1% used blinding for outcome appraisers, which carry moderate bias risk. Finally, 80.4% of the included studies described losses in follow-up and exclusions, and 65.2%reported randomization in subject assignment, which carry moderate risk of bias. |
| Martín-Valero R. et al. 2020 | PEDro scale | Mean PEDro score of 6.375 out of 10. We found seven studies with level 1 evidence (good; 75% [7/9]); one study had score of 5, which is considered level 2 evidence (acceptable; 12.5% [1/9]); and another study had scores of 4 or less, which is considered (poor; 12.5% [1/9]). Trials were considered of enough methodological quality if they had a score of at least 5 out of 10 points. |
| Chen TA. et al. 2022 | Cochrane Collaboration Risk of Bias Tool 2 (RoB 2) | All RCTs reported acceptable methods of randomisation, but four RCTs did not mention the process of allocation concealment. Five RCTs indicated that patients were blinded through the application of a placebo or sham exercise. Although the other two RCTs did not provide adequate blinding information, the patients were asked to visit the hospital every week to determine their compliance to prevent bias resulting from deviation from intended interventions. Moreover, four of seven studies had a low risk of bias from missing outcome data; among the remaining three, one study reported a 40% loss to follow-up because of failure to complete the outcome measurement, one study indicated a 47% loss to follow-up due to participants’ change of residence area and the presence of arboviral diseases in both groups, and one study revealed a 45% loss to follow-up due to unavailability or surgery. All trials had a low risk of bias in outcome measurement and the selection of reported results. Therefore, the overall risk of bias was low in one study and some concerns in other six studies. No study has high risk of bias. |
| Chen Y. et al 2022 | Jadad quality assessment scale (Jadad et al. 1996) | Four studies obtained 3 points on the jadad scale (high quality), and two studies obtained 4 points (high quality). |
| Dar JA. et al. 2022 | Cochrane Collaboration Risk of Bias Tool 2 (RoB 2) | Seven studies were included in which only two had low risk of bias as method of randomization process was described and the remaining five studies reported as high risk of bias as there was lack of detail in the randomization method. The five studies had low risk of bias in allocation concealment (envelope method, telephone service) one had a higher risk and in one study it was unclear. Three studies were of lower risk because of participant’s blinding while remaining four had higher risk of bias due to lack of blinding. The selective reporting data and incomplete data were of low risk in all the selected seven studies in both the domain. Five studies were of lower risk due to the placebo, whereas the two studies the risk of bias was unclear. |
| Luo Z. et al. 2022 | Cochrane Collaboration Risk of Bias tool | Most of the included studies described the details regarding allocation concealment, blinding of participants and personnel, blinding of outcome assessment, incomplete outcome data, and selective reporting. The other indexes of bias typically lacked specific descriptions in the included clinical studies. The study by Laoutaris et al. did not report in detail the Cochrane risk of bias assessment, so the level of risk was not clear for most of the assessment components. |
| Torres-Castro R. et al. 2022 | Cochrane Collaboration Risk of Bias tool | All studies had a high or unclear risk of bias in at leats one domain. The majority of studies claimed to be randomised. However, only half of them explain how the randomisation was done. Three studies reported that participants and personnel were blinded. Three studies reported that researchers and outcome assessments were blinded. Two studies had insufficient data on attrition rates. Four studies had a low risk of selective reporting; only two studies had a high risk of selective reporting. Finally, four studies had a high risk of other potential sources of bias due to poor participant compliance, sample size or baseline differences. |
| Wang Q. et al. 2022 | Cochrane Collaboration Risk of Bias tool | Three studies reported random sequence generation, five studies stated the allocation concealment process,and all of the studies were estimated to have a low risk ofreporting bias. However, for blinding of participants and personnel, the studies were all judged as high risk because the design of the study itself. Six studies specified blinding of outcome assessment. One study with a high incidence of withdrawal was considered high risk in the domain of incomplete outcome data. Meanwhile, in the domain of other biases, all the studies were deemed to have alow risk except for one study (lack of a no treatment control group). |
| Yang MX. et al. 2022 | PEDro scale | The average score of included RCTs in the PEDro scale was 6.43, ranging from 5 to 7, which indicates high quality. |
| Gutierrez-Arias R. et al. 2023 | Cochrane Collaboration Risk of Bias Tool 2 (RoB 2) | The risk of bias for the outcome maximal and functional exercise capacity was rated as “low risk” for two studies and “high risk” in the remaining five studies due to lack of information and because of the problems associated with measuring the outcome due to its effort-dependent nature and the lack of blinding of assessors. In the case of quality of life, the risk of bias was rated as “low risk” in two studies due to the control group receiving sham intervention, “some concerns” in one study due to doubts in the process of concealment of the random allocation, and “high risk” in three studies due to issues arising from the measurement of outcome associated to self-reporting and lack of blinding of participants (control group not received sham intervention). And, for dyspnea, the risk of bias was rated as “low risk” in two studies due to the control group receiving sham intervention, “some concerns” in one study due to doubts in the process of concealment of the random allocation, and “high risk” in one study due to issues arising from the measurement of outcome associated with self-reporting and lack of blinding of participants (control group not received sham intervention). For our secondary outcomes, the risk of bias was rated as “low risk” due to the control group receiving sham intervention, or “high risk” and due to the problems associated with measuring the outcome due to its effort-dependent nature and the lack of blinding of assessors. |
| Lista-Paz A. et al. 2023 | Cochrane Collaboration Risk of Bias Tool 2 (RoB 2) and PEDro scale | Nine of the 11 reports had at least one “unclear risk” domain, and five reports had at least one “high risk” domain. Seven reports had issues with the reporting data and eight with the allocation procedure. The average score for the PEDro scale was 6.8/10, considered “good” methodological quality across the studies included in the systematic review. |
| Silva de Sousa et al. 2023 | Cochrane Collaboration Risk of Bias Tool 2 (RoB 2) | All studies were judged as having an overall high risk of bias. Most studies raised some concerns regarding the randomization process; however, a majority of the studies were assessed to have a high risk of bias in the sections of deviations from the intended interventions and missing outcome data. |
| Ammous et al, 2023 | Cochrane Collaboration Risk of Bias Tool 2 (RoB 2) | The main issues we found across the studies were the lack of details about allocation concealment (randomization process), lack of blinding (measurement of the outcome, although we considered using a sham IMT equal to blinding participants), and only journal articles were available (selection of the reported results). |
